# Leveraging Automated Machine Learning for the Analysis of Global Public Health Data: A Case Study in Malaria

**DOI:** 10.3389/ijph.2021.614296

**Published:** 2021-04-13

**Authors:** Elisabetta Manduchi, Jason H. Moore

**Affiliations:** ^1^ Department of Biostatistics, Epidemiology and Informatics, University of Pennsylvania, Philadelphia, PA, United States; ^2^ Institute for Biomedical Informatics, University of Pennsylvania, Philadelphia, PA, United States

**Keywords:** automated machine learning, malaria, public health resources, genetic programming, predictive modeling

The traditional approach to modeling the determinants of an outcome of interest in epidemiology consists of employing statistical methodologies, typically regression-based analyses. An alternative, which is becoming increasingly appreciated in the field, is offered by machine learning (ML), i.e., algorithmic based strategies which take advantage of the increased computational power over the past decade. In supervised ML, a *training* dataset is used to learn a predictive model for a *target* (the ML term for the outcome, i.e., the dependent variable) from specified *features* (the ML term for the explanatory, i.e., the independent, variables). The predictive performance of the resulting model is then assessed on a hold-out *testing* dataset. One of the main appeals of ML is that it offers more flexibility in terms of the assumptions about the variables and their relationships.

ML approaches yielded improved Risk of Sepsis scores [1] and models for groundwater nitrate exposure [2], to cite a few examples. However, setting up a suitable ML pipeline for a specific task involves several steps and decisions, including data pre-processing, feature selection, feature engineering, selection of ML algorithm(s), and tuning of the algorithm hyperparameters [3]. Optimizing ML-based pipelines with respect to predictive performance is therefore labor intensive and requires considerable domain expertize. An exciting development in the field consists of methods which assist (potentially non-expert) users in the design and optimization of ML pipelines. These methods are termed automated machine learning (AutoML) [4]. The Tree-based Pipeline Optimization Tool (TPOT) [5, 6] is an AutoML which employs genetic programming [7] to explore pipelines consisting of combinations of feature selection, feature transformation, and classification or regression steps and recommends the pipeline with the best performance. Good practice is to then assess the predictive performance of this pipeline on a separate hold-out testing dataset. TPOT offers a choice of several metrics to assess pipeline performance. In the application illustrated below we chose accuracy, the proportion of correctly classified individuals.

TPOT has been successfully used in biomedical applications including genetics [5], metabolomics [8, 9], transcriptomics [10, 11], and toxicogenomics [10]. Here we describe an application to infectious disease epidemiology leveraging data from ClinEpiDB, a resource aimed at advancing global public health by facilitating the exploration and analysis of epidemiological studies [12]. We selected the India International Centers of Excellence in Malaria Research (ICEMR) cross-sectional study of malaria in different transmission settings [13]. In the traditional regression-based analyses on this dataset reported in [14], the independent variables were age bin (<5, 5–14, 15+), gender, a history of travel in the two weeks preceding the survey visit, a history of malaria in the past year, antimalarial use in the two weeks preceding the visit, reported use of repellent, and whether the visit occurred during the rainy season. There were five outcomes of interest (malaria detected by PCR, or by microscopy, submicroscopic/symptomatic/asymptomatic malaria) and analyses were stratified by site in India (Chennai/Nadiad,/Rourkela) and by *Plasmodium* species (*falciparum*/*vivax*/any), for a total of 45 analyses. We analyzed the same stratified combinations of targets and features using TPOT. As an illustration, we describe our TPOT results for one of these combinations, namely the analysis on individuals from Nadiad where the (binary) target is whether the individual had malaria (any species) detected by microscopy (58 cases, 793 controls). None of the features listed above was identified as significantly associated to the outcome in the regression analyses reported in [14]. We used TPOT to explore three increasingly complex types of pipelines: i) a single regularized logistic regression (LR) step, or ii) a combination of feature selection, feature transformation, and LR steps, or iii) a combination of feature selection, feature transformation and classification steps. For each of these three types, we ran TPOT 50 times with different random splits of the input data into training (75%) and hold-out testing (25%) portions. Moreover, to mitigate the effect of the high imbalance between number of cases and controls, in each run we randomly undersampled the controls to equal the number of cases prior to the random split. Figure 1 summarizes the results, where each point represents one of the 50 runs of TPOT exploring pipelines of the type indicated by its color. On the *y-*axis the accuracy on the testing set of the TPOT-optimized pipeline for that run is indicated. The Kruskal-Wallis test did not detect a significant difference in the three distributions. For this dataset, simple pipelines of type (i) achieve good microscopic malaria prediction accuracy on average (mean = 0.68), when the regularized LR hyperparameters are tuned. Models of type (i) generalize those used in [14], and are easily interpretable. The best accuracy across these 50 TPOT runs is 0.86. On the other hand, the best accuracies across type (ii) and (iii) runs are ∼90%. There is a tradeoff between interpretability and complexity. Figure 2 depicts the architecture of the best pipeline of type (iii). This has a higher accuracy than the best LR pipeline but is quite complex and it would have unlikely been discovered without an AutoML approach. The most relevant features in this pipeline, in terms of driving the predictive model, were gender and antimalarial use, based on permutation importance, which is a standard ML method to aid in model interpretability, described at https://eli5.readthedocs.io/en/latest/blackbox/permutation_importance.html#eli5-permutation-importance.

**FIGURE 1:**
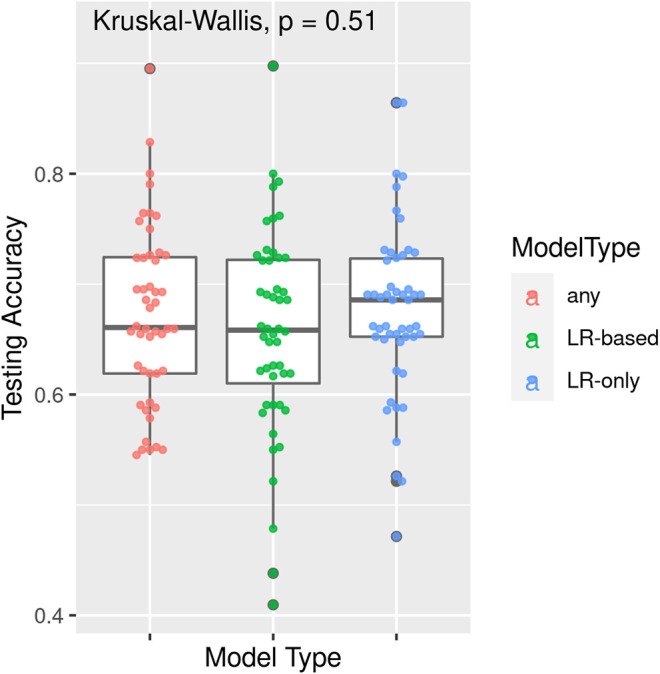
Results of 50 TPOT runs for each of model types (i) a single regularized logistic regression step, (ii) a combination of feature selection, feature transformation, and regularized logistic regression steps, and (iii) a combination of feature selection, feature transformation and classification steps. Each point corresponds to a run and its *y*-coordinate indicates the accuracy of the pipeline optimized in that run over the hold-out testing dataset. Data are from the ICEMR cross-sectional study of malaria, India, 2012–2014.

**FIGURE 2:**
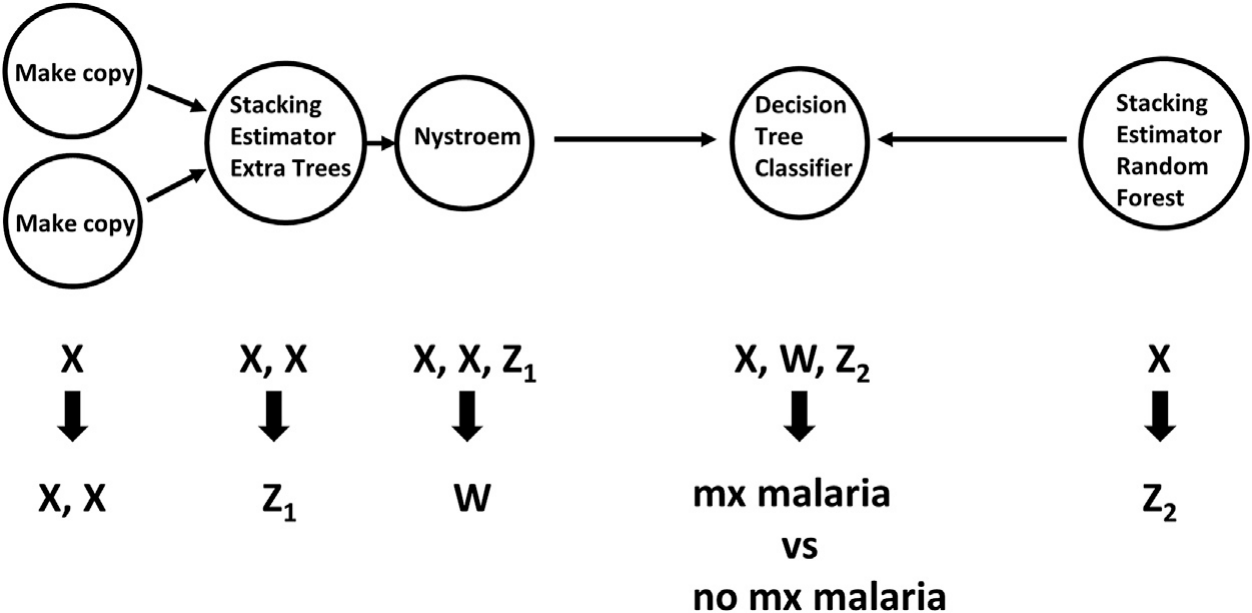
Workflow of feature selection, feature transformation and classification steps for the pipeline of type (iii) with the best accuracy. Arrows indicate step outputs which are fed as inputs of subsequent steps. When exploring pipeline space, currently the Tree-based Pipeline Optimization Tool can choose among five feature selection, 14 feature transformation and 15 classification algorithms. The node labels in this graph indicate the algorithms selected in this pipeline. Nystroem is a feature transformation algorithm; Extra Trees, Decision Tree and Random Forest are classifiers. The Stacking Estimator adds to its input features the results of applying the indicated classifier to those features. The Tree-based Pipeline Optimization Tool tuned hyperparameter values for each step are not shown for visual simplicity. Data are from the ICEMR cross-sectional study of malaria, India, 2012–2014.

This example underscores the utility of AutoML approaches in epidemiology, especially those offering to non-expert users the ability to specify the type of pipelines to explore, from very simple to very complex, at the same time leaving the heavy lifting to the AutoML. We add that the most recent extension of TPOT enables covariate adjustments [10] which in some epidemiology settings is crucial. Embedding AutoML tools within epidemiology platforms like ClinEpiDB would empower users to directly perform sophisticated analyses, accelerating the benefits derived from these public health resources.

## Data Availability

Publicly available datasets were analyzed in this study. This data can be found here: https://clinepidb.org/ce/app/record/dataset/DS_a5c969d5fa.
